# Human adipose-derived stem cells enriched with VEGF-modified mRNA promote angiogenesis and long-term graft survival in a fat graft transplantation model

**DOI:** 10.1186/s13287-020-02008-8

**Published:** 2020-11-19

**Authors:** Fei Yu, Nevin Witman, Dan Yan, Siyi Zhang, Meng Zhou, Yan Yan, Qinke Yao, Feixue Ding, Bingqian Yan, Huijing Wang, Wei Fu, Yang Lu, Yao Fu

**Affiliations:** 1 Department of Ophthalmology, Ninth People’s Hospital, School of Medicine, Shanghai Jiao Tong University, Shanghai, 200011 China; 2 Shanghai Key Laboratory of Orbital Diseases and Ocular Oncology, Shanghai, 200011 China; 3 Department of Cell and Molecular Biology, Karolinska Institute, Stockholm, Sweden; 4 Department of Plastic Surgery, Ninth People’s Hospital, School of Medicine, Shanghai Jiao Tong University, Shanghai, 200011 China; 5 Department of Pediatric Cardiothoracic Surgery, Shanghai Children’s Medical Center, School of Medicine, Shanghai Jiao Tong University, Shanghai, 200127 China; 6 Institute of Pediatric Translational Medicine, Shanghai Children’s Medical Center, School of Medicine, Shanghai Jiao Tong University, Shanghai, 200127 China

**Keywords:** hADSCs, modVEGF, Fat transplantation, Angiogenesis, Graft survival

## Abstract

**Background:**

Fat grafting, as a standard treatment for numerous soft tissue defects, remains unpredictable and technique-dependent. Human adipose-derived stem cells (hADSCs) are promising candidates for cell-assisted therapy to improve graft survival. As free-living fat requires nutritional and respiratory sources to thrive, insufficient and unstable vascularization still impedes hADSC-assisted therapy. Recently, cytotherapy combined with modified mRNA (modRNA) encoding vascular endothelial growth factor (VEGF) has been applied for the treatment of ischemia-related diseases. Herein, we hypothesized that VEGF modRNA (modVEGF)-engineered hADSCs could robustly enhance fat survival in a fat graft transplantation model.

**Methods:**

hADSCs were acquired from lipoaspiration and transfected with modRNAs. Transfection efficiency and expression kinetics of modRNAs in hADSCs were first evaluated in vitro. Next, we applied an in vivo Matrigel plug assay to assess the viability and angiogenic potential of modVEGF-engineered hADSCs at 1 week post-implantation. Finally, modVEGF-engineered hADSCs were co-transplanted with human fat in a murine model to analyze the survival rate, re-vascularization, proliferation, fibrosis, apoptosis, and necrosis of fat grafts over long-term follow-up.

**Results:**

Transfections of modVEGF in hADSCs were highly tolerable as the modVEGF-engineered hADSCs facilitated burst-like protein production of VEGF in both our in vitro and in vivo models. modVEGF-engineered hADSCs induced increased levels of cellular proliferation and proangiogenesis when compared to untreated hADSCs in both ex vivo and in vivo assays. In a fat graft transplantation model, we provided evidence that modVEGF-engineered hADSCs promote the optimal potency to preserve adipocytes, especially in the long-term post-transplantation phase. Detailed histological analysis of fat grafts harvested at 15, 30, and 90 days following in vivo grafting suggested the release of VEGF protein from modVEGF-engineered hADSCs significantly improved neo-angiogenesis, vascular maturity, and cell proliferation. The modVEGF-engineered hADSCs also significantly mitigated the presence of fibrosis, apoptosis, and necrosis of grafts when compared to the control groups. Moreover, modVEGF-engineered hADSCs promoted graft survival and cell differentiation abilities, which also induced an increase in vessel formation and the number of surviving adipocytes after transplantation.

**Conclusion:**

This current study demonstrates the employment of modVEGF-engineered hADSCs as an advanced alternative to the clinical treatment involving soft-tissue reconstruction and rejuvenation.

## Background

There is currently a clinical need for novel employable techniques to reconstruct congenital and acquired soft tissue defects in an efficient and safe manner. Fat grafting has become a preferred clinical treatment for soft-tissue augmentation and reconstruction, as it is a source of autologous cells and is easily obtained through liposuction procedures. Autologous fat is currently employed for a variety of cosmetic or reconstructive indications including sequelae of radiation therapy, breast augmentation, trauma, facial rejuvenation, and orbital fracture [1, 2]. Despite the advantages of the wide sources of fat and its non-immunogenic properties, the long-term poor outcomes of fat grafting caused by high absorption rate are critical drawbacks and a major limitation [3, 4]. As reported, the survival rate of grafted fat ranges from 20 to 80% [5, 6]. The resulting volume reduction observed in fat grafts is met with adipocyte apoptosis and necrosis, fibrocystic invasion, and eventually loss of fat volume and function, which is mainly caused by insufficiency and untimely revascularization [7].

In this regard, numerous approaches have been applied to fat grafting procedures involving co-treatment regimes in the form of recombinant proteins, biomaterials, and gene modifications of stem cells in hopes to stimulate pro-angiogenesis/vascularization of the grafts [8, 9]. The vast majority of these approaches have been met with contradictory outcomes. More recently, cell-assisted fat transfer employing human adipose-derived stem cells (hADSCs) has been shown to significantly enhance fat graft survival [10, 11]. As reported, hADSCs not only have the characteristics of rapid amplification, non-immunogenicity, and pluripotent differentiation potential, but also are easily and abundantly accessed via standard liposuction procedures [12, 13]. However, the full potential of hADSCs in regenerative research has not yet been realized, as many researchers continue to engineer hADSCs to more effectively enhance aspects of regenerative medicine [14–16].

Synthetic chemically modified mRNAs (modRNAs) are gaining praise as a novel modality for forced, transient gene expression [17–19]. Unlike DNA and viral plasmids/vectors, modRNAs are expressed as potent and transient bursts of gene expression, capable of delivering an efficient and dose-controlled level of protein, while exhibiting minimal immunogenicity [17]. Several studies employing different myocardial infarction models revealed that intramyocardial injection of modRNA encoding the vascular endothelial growth factor A_165_ (VEGF-A_165_) gene-induced cardiac regeneration [20, 21]. Moreover, a recent clinical study also suggested that intradermal injection of VEGF modRNA (modVEGF) was well tolerated in men with type 2 diabetes mellitus (T2DM), where the delivery induced local functional VEGF protein expression leading to increased dermal blood flow [22]. More recently, Yu et al. applied a cell-mediated modVEGF delivery system employing human fibroblasts to treat critical limb ischemia (CLI) [23]. In this aforementioned study by Yu et al., a cell-mediated approach for protein delivery through modRNAs revealed enhanced tissue regeneration when compared to the naked delivery of modVEGF alone by extending the window of expression and increasing the therapeutic potency of the protein, ultimately giving rise to healthier blood vessels. Worth mentioning, VEGF is expressed at subtle levels in hADSCs, where it is thought to play a role in increasing the survival of fat grafts [14, 24–26]. Taken together, we proposed that a stem cell-proangiogenic factor hybrid therapy might be beneficial in enhancing the angiogenic potential of hADSCs and could significantly improve fat retention post-transplantation.

Herein, for the first time, we combined modVEGF with hADSCs from patient lipoaspirates to improve survival of fat transplantation. We report that, compared to hADSCs alone, modVEGF-engineered hADSCs more efficiently enhanced fat grafting through mechanisms involving neo-angiogenesis, cell proliferation, and simultaneously alleviated levels of fibrosis, apoptosis, and necrosis in the ischemic setting. Furthermore, modVEGF-engineered hADSCs were met with preserved viability and enhanced differentiation into vessels and adipocytes in situ. Thus, the combination of hADSCs with modRNAs could be an advanced course of action for regenerative therapies, including fat transplantation for tissue augmentation and reconstructive surgery.

## Materials and methods

### Isolation and culture of human adipose-derived stem cells (hADSCs)

The adipose tissue was derived from healthy female patients whom underwent liposuction or autologous fat transplantation for cosmetic purposes. The Ethics Committee of Shanghai Ninth People’s Hospital approved the study. Standard liposuction techniques were employed as previously reported, and hADSCs were isolated and cultured following reported protocols [8, 27]. Briefly, fat tissue for grafting and hADSC extraction was separated from lipoaspirates via rinsing with PBS, centrifugation, and aspiration. The fat tissue was digested with 0.2% collagenase A (Roche, Manheim, Germany) at 37 °C for 1 h, followed by centrifugation at 1500 rpm for 10 min. The cell pellet was resuspended in Dulbecco’s modified Eagle’s medium (DMEM) (HyClone, GE Healthcare, Little Chalfont, UK) supplemented with 10% fetal bovine serum (FBS) (Invitrogen, California, USA) and 1% penicillin-streptomycin antibiotic (Gibco, Carlsbad, CA, USA) and then seeded on 10-cm dishes and sub-cultured using trypsin (Gibco, Carlsbad, CA, USA) when the cells reached 80–90% confluence. hADSCs were employed between passages 3 and 5 for all studies described.

### modRNA synthesis and formulation

modRNA was synthesized in vitro using T7 RNA polymerase-mediated transcription from a linearized DNA template, which incorporated generic 5′ and 3′UTRs and a poly-A tail, as previously described [23, 28, 29]. During the in vitro transcription reaction, we fully replaced uridine with N1-methylpseudouridine. RNA was purified using Ambion MEGAclear spin columns and treated with Antarctic phosphatase (New England Biolabs) for 30 min at 37 °C to remove residual 5′-phosphates. The RNA was repurified and quantified by Nanodrop (Thermo Scientific, Waltham, MA, USA), and then, modRNA was resuspended in 10 mM Tris HCl, 1 mM EDTA at 1 μg/μL for use. Open reading frame sequences used for modRNA production were the same as previously described for EGFP, firefly Luciferase, and human VEGF-A_165_ [20].

### modRNA transfection

In order to transfect hADSCs with modRNAs, MessengerMAX (Invitrogen, California, USA) transfection reagent was employed. For in vitro experiments, modRNAs and transfection reagents were first diluted separately in Opti-MEM basal medium (Invitrogen, California, USA) and incubated for 5 min at room temperature (RT). Afterwards, the two mixes were pooled together and incubated for 15 min to generate modRNA-lipid complexes. modRNA-lipid complexes were exposed to cells for 4 h, after which the medium was completely replaced with cell culture media or removed to collect cells. Specifically, for lipo-complexing, 2 μL of MessengerMAX transfection reagent was used per 1 μg modRNA to transfect per 1 × 10^5^ hADSCs in the in vitro and in vivo experiments.

For evaluating GFP modRNA (modGFP) expression kinetics in hADSCs, the transfection efficiency and mean fluorescence intensities of modGFP were recorded at 4, 8, 16, 24, and 48 h post-transfection, by C6 flow cytometry (Beckman Coulter, CA, USA).

### Expression of modVEGF in vitro

#### Real-time PCR

Total RNA from cultured hADSCs were extracted by TRIzol reagent (Invitrogen, California, USA) at 24 h following transfection, and the reverse transcription was employed using PrimeScript RT reagent kit (Takara Bio Inc., Otsu, Japan) as previously reported [14]. Real-time PCR was performed using Power SYBR Green PCR Master Mix (Applied Biosystems, Foster, CA, USA) according to the protocol. The following primers were used: VEGF-A, forward primer, 5′-TTGCAGGTTGGTT CCCAGAGG-3′ and reverse primer, 5′-TCGGCTTGTCACATCTGAGGG-3′; β-actin, forward primer, 5′-GGGACCTGACTGACTACCTC-3′; and reverse primer, 5′-TCAT ACTCCTGCTTGCTGAT-3′. qPCR detection was carried out on a 7500 Real-Time PCR Detection System (Thermo Scientific, Waltham, MA, USA), and relative expression was determined using the 2-∆∆CT method.

#### Western blot assay

Western blot analysis was conducted as previously described [30]. Briefly, total protein from cell lysates was extracted using lysis buffer (Thermo Scientific, Waltham, MA, USA) and the concentration was measured using a BCA kit (Pierce, Rockford, IL, USA). A 50-mg protein was loaded onto SDS-PAGE and then transferred to polyvinylidene difluoride membrane. After blocking with 5% skimmed milk, the membranes were then incubated with primary antibodies against VEGF-A (ab52917; Abcam, MA, USA) and β-actin (ab8226; Abcam, MA, USA) at 4 °C overnight. After incubation with DyLightTM680-conjugated secondary antibodies (Sigma, St. Louis, MO, USA), the protein expression levels were quantified using an Odyssey V 3.0 image scanner (LI-COR, Lincoln, Nebr., USA).

#### Enzyme-linked immunosorbent assay (ELISA)

For measuring the expression kinetics of modVEGF, the cell culture supernatant was collected at specified time points (4, 8, 16, 24, 48, 72, 96, 120 h after transfection) and concentrations of human VEGF-A protein were quantified using ELISA (R&D Systems, Inc., Minneapolis, MN, USA) according to the manufacturer’s instructions. The optical density values of absorbance were measured on a microplate reader (ELX800, BioTek, USA).

### Characteristics of hADSCs

#### Proliferation of hADSCs

hADSCs were seeded in a 96-well plate at a concentration of 2 × 10^3^ cells/well, each group containing six counter holes. After the treatments, the Cell Counting Kit-8 (CCK8) (Dojindo, Kumamoto, Japan) was used to conduct a cell proliferation assay at different time points. An optical density at 450 nm was recorded using a microplate reader (ELX800, BioTek, USA). Average data were presented as percentages of the O.D. values relative to the control group.

#### Migration of hADSCs

Migration assay was performed using 24-well polycarbonate membrane cell culture plate inserts with 8-μm-pore size (SPL Life Sciences Co., Ltd., Korea). At first, hADSCs in each group were cultured for 24 h post-transfection. After trypsinization, 2 × 10^4^ cells were seeded on the top chambers of the transwell inserts. The top chambers contained 200 μL serum-free medium, and the bottom chambers contained 400-μL of 10% serum medium. After 12 h of incubation, cells passed through the membrane were dyed by crystal violet and counted to calculate the means of triplicate experiments.

#### Phenotypic profile of hADSCs

The phenotypic profile of hADSCs was determined by flow cytometry as previously described [31]. Briefly, approximately 5 × 10^4^ cells were incubated with fluorescence-conjugated antibodies, which were all obtained from BD Biosciences for 30 min at RT. Quantitative analysis was performed using C6 flow cytometry (Beckman Coulter, CA, USA), and results were analyzed by FlowJo software (Tree Star Inc., Ashland, OR, USA).

#### Multipotent differentiation of hADSCs

The trilineage differentiation potentials of hADSCs were tested as performed in a previous article [32]. All chemicals were purchased from Sigma, St. Louis, MO, USA. According to instructions, pretreated-hADSCs were cultured separately in AdipoDiff, OsteoDiff, and ChondroDiff Medium for 21 days, followed by Oil Red O, Alizarin Red S, and Alcian Blue stainings. Images of differentiated hADSCs were obtained and evaluated.

### Matrigel plug assay

Six-week-old male BALB/c-nude mice in our study were purchased from the Animal Laboratory, Shanghai Ninth People’s Hospital, Shanghai Jiao Tong University School of Medicine, Shanghai, China. All experimental protocols were approved by the Animal Care and Experiment Committee of the Shanghai Ninth People’s Hospital. Three mice were used for the implantation of Matrigel plugs as previously described [23]. In brief, ice-cold Matrigel (BD Biosciences, San Jose, CA, USA) (200 μL per plug) was mixed with 50 μL PBS or hADSCs (1 × 10^6^ cells in 50 μL PBS) and then injected subcutaneously into the dorsal region of mice (each mouse received three plugs in total from the different groups). The plugs of the PBS group contained Matrigel matrix mixed with only PBS; the plugs containing Matrigel matrix mixed with hADSCs pretreated with either Luciferase modRNA (modLuc) or modVEGF were termed the ADSC^modLuc^ group or the ADSC^modVEGF^ group, respectively. For the ADSC^modLuc^ and ADSC^modVEGF^ groups, a total of 10 μg modRNA and 20 μL MessengerMAX reagent was used for 1 × 10^6^ cells. One week after implantation, Matrigel plugs were excised for gross and histological evaluation.

### Fat transplantation model

Nude mice models of fat grafting were constructed as previously described [33]. The mice were randomly divided into the PBS group, the ADSC^modLuc^ group and the ADSC^modVEGF^ group. In the PBS group, a mixture of 400 μL of fat with 100 μL of PBS alone was injected. In the ADSC^modLuc^ group, a mixture of 400 μL of fat with 100 μL of PBS containing 2 × 10^6^ modLuc-engineered hADSCs was injected. In ADSC^modVEGF^ group, a mixture of 400 μL of fat with 100 μL of PBS containing 2 × 10^6^ modVEGF-pretreated hADSCs was injected. Before injection, the mixture of each group was mixed well in dishes and syringes of each group were not cross-used. Thirty-six mice in total were used. As shown in Fig. 4a, each mouse received implants of three fat grafts via a 14-G needle, where each graft represented a different treatment group. Fat grafts were harvested at desired time points for further analysis. We randomly selected eighteen mice for sacrifice at 1, 2, 3, 4, 5, and 6 days after grafting to evaluate in vivo protein levels of VEGF-A and HIF1α by ELISA (R&D Systems, Inc., Minneapolis, MN, USA) (*n* = 3 each time point). The remaining eighteen mice were sacrificed at 15, 30, and 90 days post-transplantation, and the fat grafts were photographed, weighed, and underwent further histological evaluation (*n* = 6 each time point). Additionally, three mice sacrificed at 90 days received fat grafts mixed with modLuc or modVEGF-engineered hADSCs that were first subjected to CM-DiI labeling (Invitrogen, California, USA). CM-DiI labels were performed as the instruction described.

### Histological staining

The Matrigel plugs and fat grafts were paraffin-embedded and cut into 6-μm-thick sections. Hematoxylin & eosin (H&E) staining was performed, and fat vacuoles, identifying the adipocytes larger than 120 μm in diameter, were evaluated as previously described [34]. To evaluate collagen accumulation, indicating fibrosis, Masson trichrome (Sigma, St. Louis, MO, USA) stainings were performed according to the manufacturer’s instructions.

For immunohistological staining, tissue sections were probed with primary antibodies at 4 °C overnight, followed by incubation with a Cy™3-conjugated secondary antibody (Jackson ImmunoResearch Laboratories, Inc., PA, USA) or Alexa Fluor 488-conjugated secondary antibodies (Invitrogen, California, USA). The following is a list of the primary antibodies used for immunohistochemistry: anti-VEGF-A antibody (ab52917; Abcam, MA, USA), anti-CD31 antibody (ab182981; Abcam, MA, USA), anti-Human Nuclear Antigen (ab191181; Abcam, MA, USA), anti-Perilipin antibody (ab61682; Abcam, MA, USA), anti-α-SMA antibody (ab21027; Abcam, MA, USA), anti-Ki67 antibody (ab92742; Abcam, MA, USA), and anti-TNF-α antibody (ab1793; Abcam, MA, USA). Terminal deoxynucleotidyl transferase-mediated d-UTP nick end labeling (TUNEL) (Roche, Manheim, Germany) staining was performed following the manufacturer’s instructions. Photomicrographs were performed using a confocal microscope (Leica microsystems, Heidelberg, Germany). Five random fields were selected for each sample (*n* = 3 each time point). All measurements were performed with Image-Pro Plus software (Media Cybernetics, MD, USA).

### Statistical analysis

The data are presented as the mean ± standard deviation. *p* values were analyzed using a one-way analysis of variance (ANOVA) followed by Tukey’s test (GraphPad Software, San Diego, CA, USA). Statistical significance is denoted by *p* < 0.05.

## Results

### Transfection of hADSCs with modified mRNAs (modRNAs) are well tolerated

**Fig. 1:**
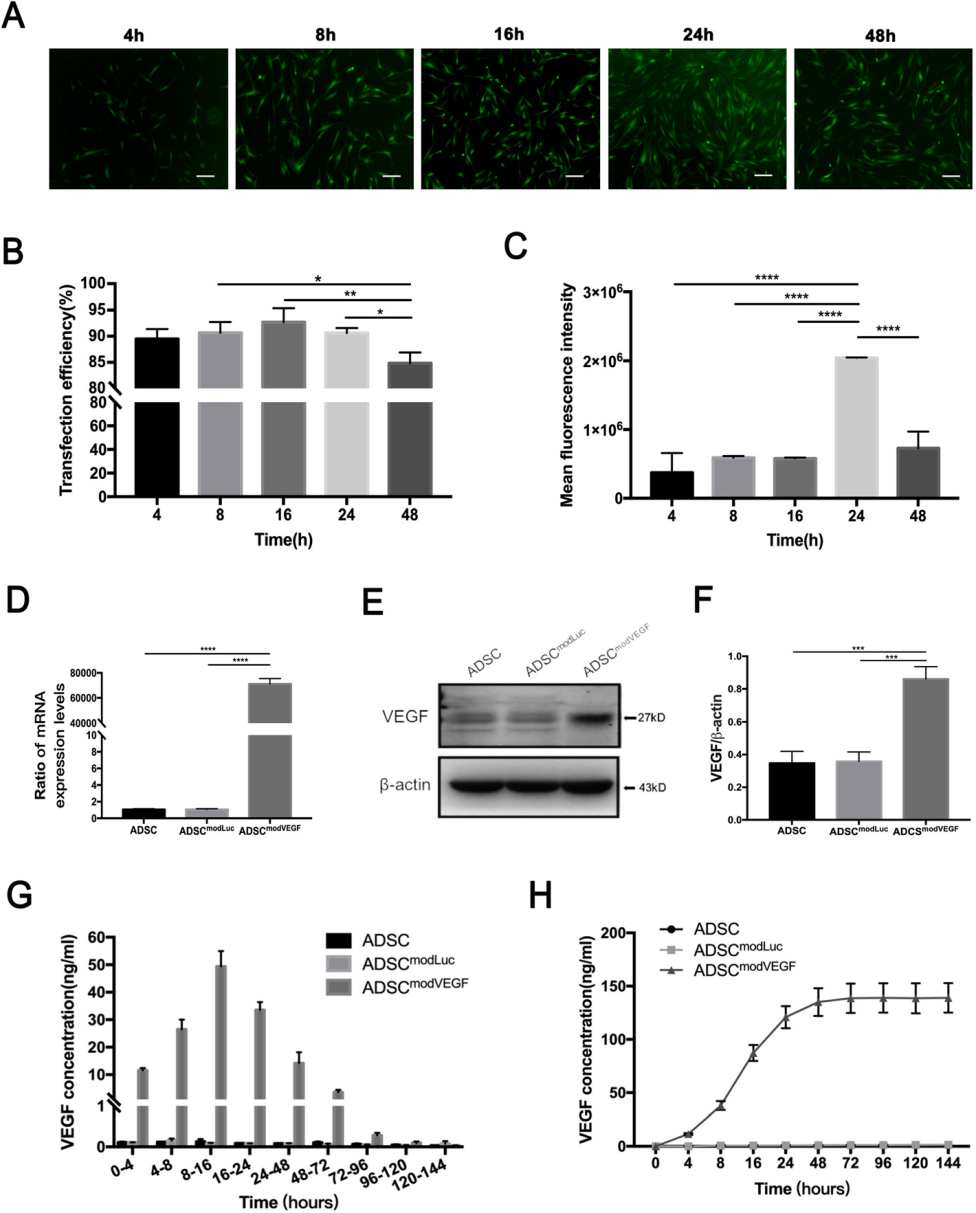
Efficacy and kinetics of modRNA transfection in hADSCs. **a**–**c** Transfection efficiency and the expression kinetics of modGFP in hADSCs. **a** Representative images depicting GFP signal in hADSCs at 4, 8, 16, 24, and 48 h post-transfection. **b** Flow cytometry analysis of transfection efficiency at 4, 8, 16, 24, and 48 h post-transfection. **c** Flow cytometry analysis of mean fluorescence signal intensity at 4, 8, 16, 24, and 48 h post-transfection. **d**–**f** Expression levels of **d** VEGF mRNA and **e**–**f** VEGF protein at 24 h post-transfection. **g**–**h** Kinetics of **g** newly produced and **h** cumulative VEGF protein concentrations periodically monitored for several days following transfection of modVEGF in hADSCs. Scale bar = 100 μm. Error bars showed means ± SD. (*n* = 3; *p† < 0.05, **p† < 0.01, ***p† < 0.001, ****p† < 0.0001)

In order to determine the transfection efficiency and kinetics of modRNAs in hADSCs, we transfected hADSCs with modRNA encoding a GFP reporter construct (modGFP). We found that hADSCs were highly tolerant of modRNA transfections as we demonstrated the transfection efficiency of modGFP in hADSCs as high as 92.2% ± 2.7% at 16 h post-transfection (Fig. 1a, b and Figure S1). The highest mean fluorescence intensity signal of the GFP protein was recorded at 2 × 10^6^ at 24 h after transfection (Fig. 1c).

In order to confirm the transfection efficiency and expression dynamics of modRNAs in hADSCs, we again separately transfected hADSCs with modRNAs encoding either the Luciferase (modLuc) or VEGF-A_165_ (modVEGF) genes and monitored transcript levels inside the cells. RT-PCR revealed more than 70,000-fold increase of VEGF mRNA expression in the modVEGF-engineered hADSC (ADSC^modVEGF^) group at 24 h after transfection, in comparison to the modLuc-engineered hADSCs (ADSC^modLuc^) group (Fig. 1d). Using western blot analysis, we demonstrated that the intracellular levels of VEGF protein in the ADSC^modVEGF^ group expressed two-fold more VEGF protein 24 h after transfection than the two control groups, confirming the translation of the modRNA (Fig. 1e, f). Moreover, VEGF mRNA and protein levels did not differ between the untransfected (ADSC) group and the ADSC^modLuc^ group (Fig. 1d–f).

As VEGF is a secreted ligand, we sought to detect the levels of newly produced and accumulated VEGF protein levels in the cell culture media following transfection in hADSCs. We found that VEGF protein concentrations were significantly higher in the medium of the ADSC^modVEGF^ group from 4 to 144 h post-transfection when compared to the two control groups (Fig. 1g, h).

### Characteristics of hADSCs transfected with modRNAs

**Fig. 2:**
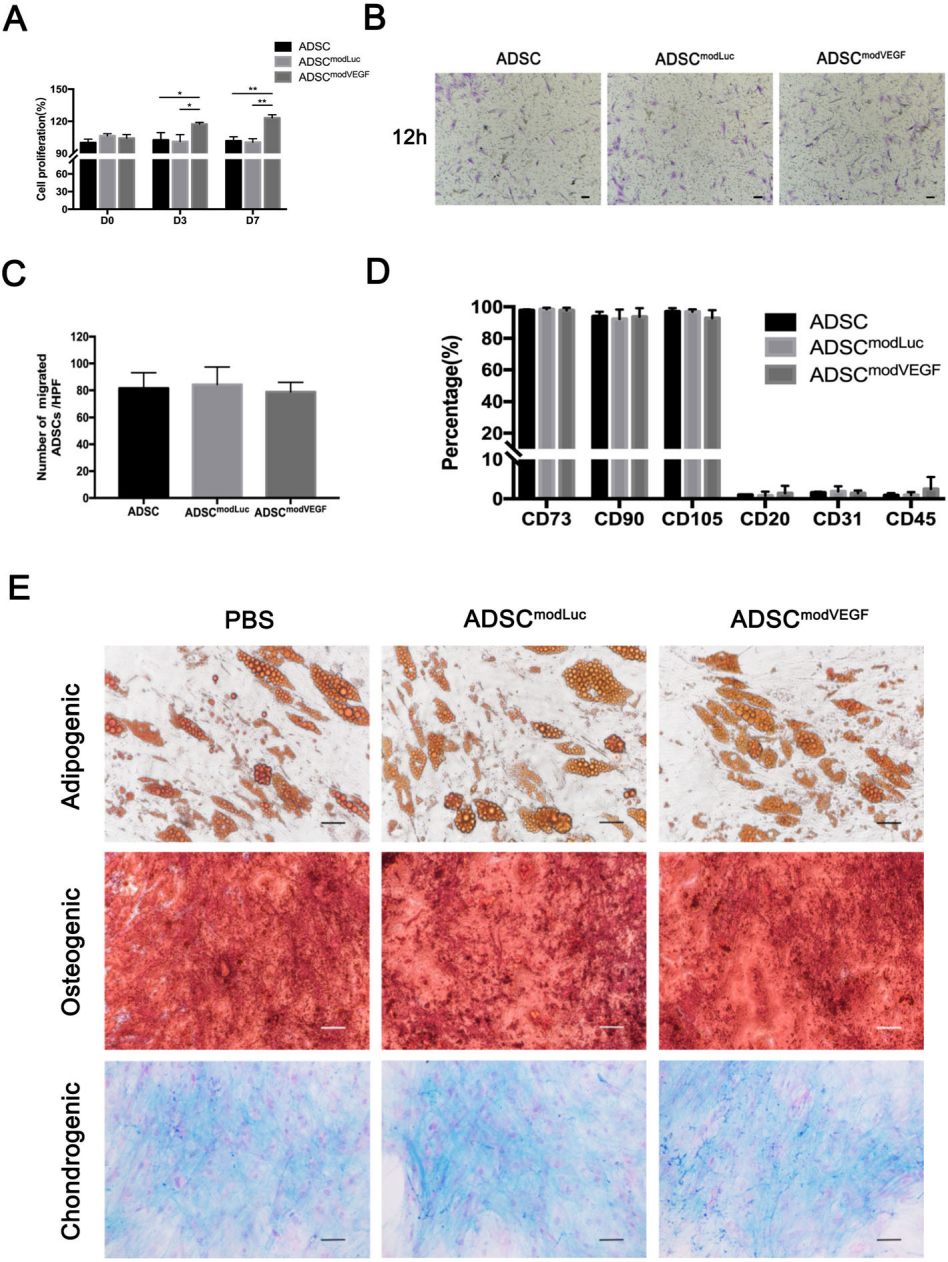
Multipotent differentiation potential and characterization of isolated and mRNA-engineered hADSCs in vitro. **a** The proliferation capability of hADSCs was assessed using cell count kit, and the percentage of optical density values relative to the control was calculated. (*n* = 6; *p† < 0.05, **p† < 0.01). **b**, **c** The migration potential of hADSCs was evaluated using transwell assay, and the number of migrated cells dyed with crystal violet (at 12 h post-seeding) was quantified. **d** Cell surface marker expression of hADSCs was analyzed at 24 h post-transfection. **e** The multipotent capacity of hADSCs following modRNA transfections was tested by inducing adipogenic, osteogenic, and chondrogenic differentiations. Successful differentiations of the lineages were detected and analyzed using Oil Red O, von Kossa, and Alcian Blue stainings, respectively. Scale bars = 50 μm. Error bars showed means ± SD. (*n* = 3; *p† < 0.05)

In order to evaluate the effects of modVEGF on hADSCs, we characterized the levels of cell proliferation, migration, and differentiation capabilities post-transfection. Using the CCK8 assay, we showed that modVEGF could promote the proliferation of hADSCs to some extent (Fig. 2a). However, transwell assays revealed no differences in migration capability between the three groups (Fig. 2b, c). By evaluating the marked surface expression of stromal markers, we discovered that hADSCs maintained their “stemness” at 24 h after modRNA-transfection. As identified by the Mesenchymal and Stem Cell Committee of the International Society for Cellular Therapy (ISCT), our treated and untreated hADSCs positively expressed stromal markers CD73, CD90, and CD105 and negatively expressed hematopoietic and endothelial markers CD20, CD31, and CD45 (Figure S2 and Fig. 2d) [35]. Furthermore, hADSCs exhibit multipotent plasticity in vitro. Therefore, we employed a trilineage differentiation assay, which revealed that modRNA treatments did not negatively inhibit/alter the adipogenic, osteogenic, and/or chondrogenic differentiation capacities of the hADSCs (Fig. 2e).

### Confirming functional in vitro secretion of VEGF protein from modVEGF-engineered hADSCs

In order to ensure that the VEGF protein secreted by modVEGF-engineered hADSCs was functionally active, the conditioned medium was applied to human umbilical cord vein cells (HUVECs). We quantitated levels of HUVEC proliferation and migration, as well as tube formation when cultured in hADSC-modVEGF conditioned media. We showed conditioned medium from modVEGF-engineered hADSCs could improve cell proliferation and migration more significantly than conditioned medium from hADSCs transfected with modLuc (Figure S3A-E). Based on a tube formation assay, we revealed more tubular structures were formed and significantly more junctions had formed in HUVECs cultured in the ADSC^modVEGF^ conditioned media (Figure S3F-H). Interestingly, subtle increases in the endothelial migration and proliferation of HUVECs were seen in the ADSC^modLuc^ group when compared to the control group. As hADSCs secrete low levels of chemokines and growth factors that enhance certain aspects of cell growth and proliferation, as previously reported, this finding was not completely unexpected (Figure S3) [26]. Since modLuc is inert but did not hinder beneficial effects seen from the hADSCs, we employed this treatment group as a continued control throughout our study.

### Proangiogenic, anti-apoptotic, and pro-proliferative effects of modVEGF-engineered hADSCs in an in vivo Matrigel plug assay

**Fig. 3:**
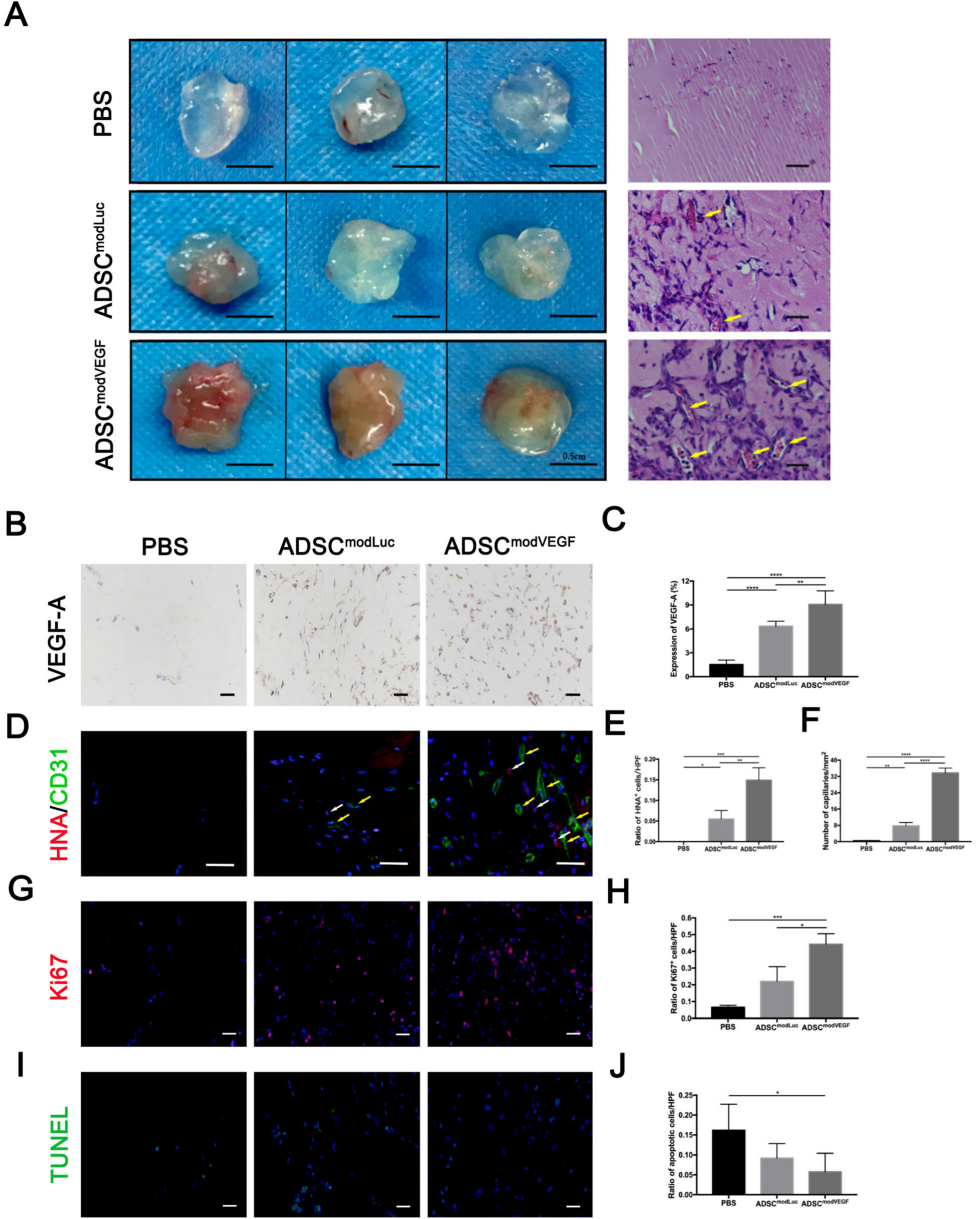
hADSCs engineered with modVEGF promotes angiogenesis in an in vivo Matrigel plug assay. Matrigel plugs harvested at 1-week post-implantation were assessed. **a** Representative gross morphological assessment (left) and microphotographs (right) of hematoxylin & eosin (H&E) stainings of extracted plugs. Yellow arrows indicate new blood vessel formation within the plug. **b** Representative photomicrographs of Matrigel plugs stained for VEGF detection. **c** The percentage of VEGF expression per area was calculated. **d** Representative photomicrographs of Matrigel plugs stained for human nuclei antibody (HNA) and CD31. White arrows partially indicate the localization of hADSCs detected with HNA. Yellow arrows partially indicate CD31^+^ blood vessels. **e**, **f** Ratio of HNA^+^ cells (**e**) and the number of CD31^+^ capillaries were quantified (**f**). **g** Representative images depicting levels of proliferating hADSCs in plugs using Ki67 stainings. **h** The ratio of Ki67^+^ cells was quantified. **i** Representative TUNEL stainings depict levels of apoptotic hADSCs within the Matrigel plugs. **j** The ratio of apoptotic cells was quantified. Error bars showed means ± SD. Scale bar = 20 μm. (*n* = 3, *p† < 0.05, **p† < 0.01, ***p† < 0.001, ****p† < 0.0001)

In order to further explore the potential pro-angiogenic, pro-proliferative, and anti-apoptotic properties of hADSCs transfected with modVEGF post-transplantation, we employed an in vivo Matrigel plug model. To examine the angiogenic efficacy of transfected modVEGF in hADSCs, we compared Matrigel mixtures containing modVEGF-engineered hADSCs (ADSC^modVEGF^) against Matrigel mixtures containing modLuc-engineered hADSCs (ADSC^modLuc^). We also included Matrigel mixtures with PBS as a negative control (PBS) group. By employing this assay, we sought to directly visualize if modVEGF-engineered hADSCs could induce new blood vessel formation in vivo. Matrigel mixtures were implanted into the subcutaneous space of the dorsal region in mice and were harvested after 1 week post-implantation for gross morphological observation (Fig. 3a) and histological analysis (Fig. 3b–j). By evaluating initial gross morphology, the plugs of the ADSC^modLuc^ group and the ADSC^modVEGF^ group both appeared more vascularized than the PBS group (Fig. 3a). Furthermore, the plugs in the ADSC^modVEGF^ group appeared significantly more vascularized and the plugs in whole appeared more morphologically complete when compared to the ADSC^modLuc^ group (Fig. 3a). Notably, we discovered that the Matrigel complexes containing ADSC^modVEGF^ gave rise to more abundant VEGF protein than ADSC^modLuc^ (*p* < 0.01) even at 1 week post-transplantation (Fig. 3b, c). In Fig. 3d, histological analysis revealed the spatial distribution of human nuclei (HNA) and vascular endothelial cells (CD31), where new blood vessels appeared to be in close proximity to the hADSCs. Following quantification, the number of hADSCs in the ADSC^modVEGF^ group was significantly higher than those found in the PBS and hADSC^modLuc^ groups (Fig. 3e). Quantitative assessment of CD31^+^ stainings revealed that plugs mixed with ADSC^modVEGF^ formed significantly more capillaries than hADSC^modLuc^ (*p* < 0.0001) (Fig. 3f). The ADSC^modVEGF^ group induced better angiogenic activity from intrinsic tissue than the ADSC^modLuc^ group, probably due to the increased number of surviving hADSCs and more potent levels of secreted VEGF protein. In order to monitor cell viability in vivo, the levels of proliferation and cell death were analyzed by employing anti-ki67 stainings and TUNEL assays (Fig. 3g–j). Plugs harvested from the hADSC^modVEGF^ group presented with significantly more Ki67^+^ cells when compared to the ADSC^modLuc^ group (*p* < 0.05), indicating the modVEGF treatment enhanced cell proliferation rates of hADSCs within the plugs (Fig. 3g, h). There were also significantly fewer apoptotic cells in the ADSC^modVEGF^ group than in the PBS (*p* < 0.05) and the ADSC^modLuc^ groups, respectively (Fig. 3i, j). Taken together, these results confirm that modVEGF-engineered hADSCs better enhanced the viability of implanted hADSCs leading to better angiogenic, pro-proliferative, and anti-apoptotic activities than hADSCs alone.

### modVEGF-engineered hADSCs significantly improve fat graft survival in vivo

**Fig. 4:**
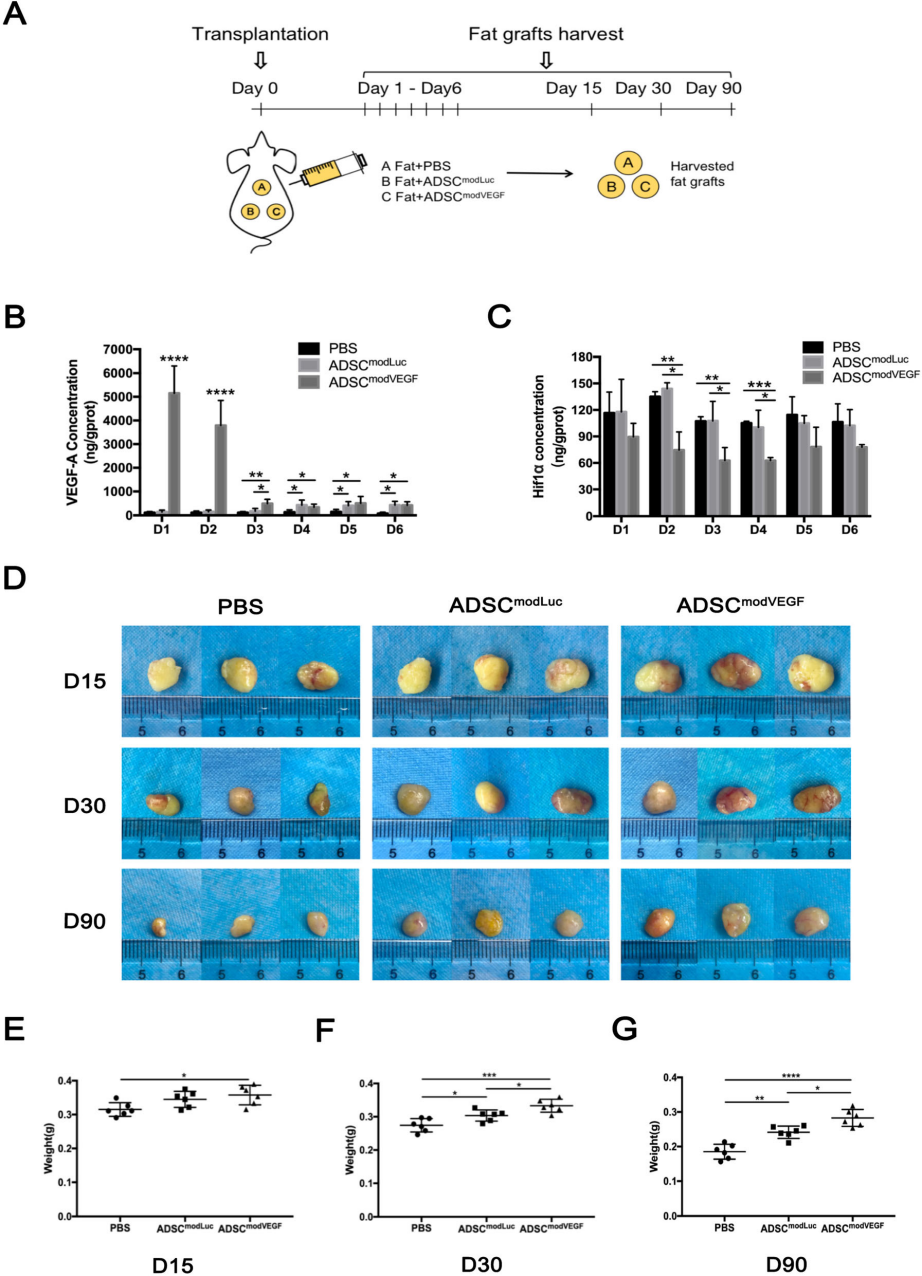
Preconditioning hADSCs with modVEGF improves fat graft survival in vivo. **a** Schematic illustration and design of the animal study. **b**, **c** Quantification of **b** VEGF-A in vivo produced protein and **c** HIF1α protein levels in fat grafts at days 1, 2, 3, 4, 5, and 6 post-transplantation. (*n* = 3) **d** Representative gross morphological images of fat grafts at 15, 30, and 90 days post-transplantation. **e**–**g** Weights of the fat grafts at 15, 30, and 90 days post-transplantation. (*n* = 6) Error bars showed means ± SD. (*p† < 0.05, **p† < 0.01, ***p† < 0.001, ****p† < 0.0001)

In order to examine the active effects of modRNA-engineered hADSCs on fat transplantation, the human fat was transplanted into nude mice together with PBS, modLuc-engineered hADSCs or modVEGF-engineered hADSCs (Fig. 4a). To determine the in vivo produced protein levels of VEGF expression stemming from modRNA-engineered hADSCs and non-treated hADSCs in fat grafts, we extracted and purified total protein from the fat implants at 1, 2, 3, 4, 5, and 6 days post-transplantation. Interestingly, we found expression levels of in vivo produced human VEGF protein in the ADSC^modVEGF^ group to be superiorly significant, nearly 5000 ng/g protein within the first 24 h post transplantation, as opposed to concentrations under 150 ng/g protein in the other groups (*p* < 0.0001) (Fig. 4b). These extremely high levels of VEGF protein production were continually detectable within the first 48 h after transplantation in the ADSC^modVEGF^ group (Fig. 4b). On the 3rd day, VEGF protein levels from the ADSC^modVEGF^ transplant group subsided; however, the protein levels remained significantly higher than the control groups (Fig. 4b). On the 4th day post-transplantation and the remainder of the quantified period (days 5 and 6 post-transplant), the levels of detected VEGF protein were similar between both the ADSC^modLuc^ and ADSC^modVEGF^ groups, which remained significantly higher than the PBS only control group (Fig. 4b). To confirm the role of the VEGF pathway further and in order to define the functional efficacy of VEGF protein in fat grafts, we examined the concentration of HIF1α protein. HIF1α protein is an upstream regulator of VEGF, whose expression levels are known to represent the degree of hypoxia in fat grafts [8, 36]. We quantified the expression levels of HIF1α across our treatment groups and controls. HIF1α levels were significantly reduced in the ADSC^modVEGF^ group during days 2–4 post-implantation, but most notably on the 4th day post-grafting (*p* < 0.001) (Fig. 4c). Taken together, these findings suggest that modVEGF-engineered hADSCs vastly enhanced therapeutic protein levels and impacted biological activities in order to regulate hypoxia-conditioning in fat grafts in vivo.

To further confirm the functional properties of modVEGF-engineered hADSCs on fat transplantation, we analyzed the gross morphology and coarse weight of grafts at 15, 30, and 90 days post-transplantation (*n* = 6 each time point). Representative images of grafts within each group are shown in Fig. 4d. The weights of fat grafts in the ADSC^modVEGF^ group were significantly higher than those in the PBS group at all investigated time points (Fig. 4e–g) and differences between the two cell-assisted fat transplant groups became evident after 2 weeks post-grafting (on the 15th day) (Fig. 4e). On the 30th day, the effects of cell-assisted fat transplants became more prominent, where significant differences were observed between both the ADSC^modLuc^ and ADSC^modVEGF^ groups when compared to PBS control (Fig. 4f). Of note, the weights of the fat grafts in the ADSC^modVEGF^ group at 90 days post-transplantation were 0.283 ± 0.024 g, which were significantly higher (*p* < 0.05) than 0.241 ± 0.018 g in the ADSC^modLuc^ group (Fig. 4g). Taken together, these results suggest that fusing modRNA technologies with stem cell-assisted fat transplants could help enhance the protective functions of hADSCs, thus further promoting heightened grafted fat survival, especially over the long term.

### Transplantation of modVEGF-engineered hADSCs enhances morphology and viability of adipocytes after grafting

**Fig. 5:**
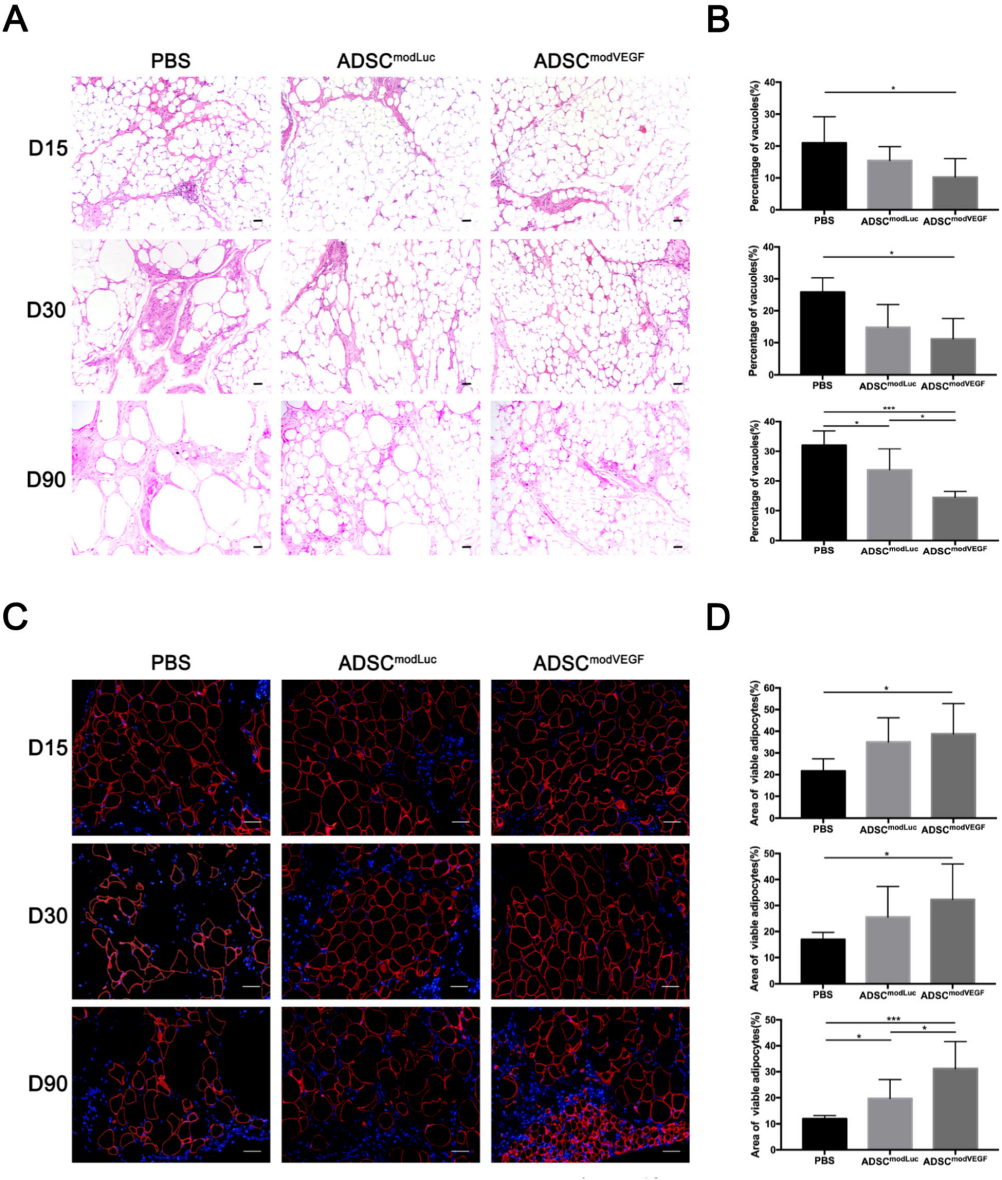
Histological analysis of viable adipocytes in fat grafts treated with native hADSCs and modRNA-engineered hADSCs. **a** Representative H&E stainings of fat grafts harvested at 15, 30, and 90 days post-transplantation. **b** Analysis of the percentage of vacuoles in the grafted fat. **c** Fat grafts stained with perilipin identifies viable adipocytes at 15, 30, and 90 days post-transplantation. **d** The percentage of the viable fat area was analyzed and quantified at 15, 30, and 90 days post-engraftment. Scale bars = 40 μm. Error bars showed means ± SD. (*p < 0.05, ***p† < 0.001)

To more deeply examine the protective potential of modVEGF-engineered hADSCs in our fat transplantation model, fat grafts were also harvested for histological studies at days 15, 30, and 90 post-transplantation. H&E stainings revealed a significant reduction in quantifiable vacuoles and connective tissue forming from the hADSC^modVEGF^ treatment group when compared to the PBS and ADSC^modLuc^ groups (Fig. 5a). Furthermore, both cell-assisted fat transplant groups, namely the ADSC^modVEGF^ as well as the ADSC^modLuc^ group revealed better anatomical morphology of adipose tissue than the PBS group, where the ADSC^modVEGF^ group gave rise to the least amount of adipocyte vacuoles (Fig. 5b). The reserved functional adipocytes of all grafts were quantitated by perilipin stainings. The morphology and area (%) of perilipin^+^ adipocytes indicated that fat grafts in the ADSC^modVEGF^ group retained significant viability over the control groups throughout the observed timeline and the differences among the three groups became apparent over time (Fig. 5c, d). From these observations, we infer that modVEGF-engineered hADSCs had the optimal effects on the survival of adipocytes and the advantages of VEGF-secreting hADSCs became gradually significant.

### modVEGF-engineered hADSCs enhance angiogenesis and proliferative activities in fat grafts in vivo

**Fig. 6:**
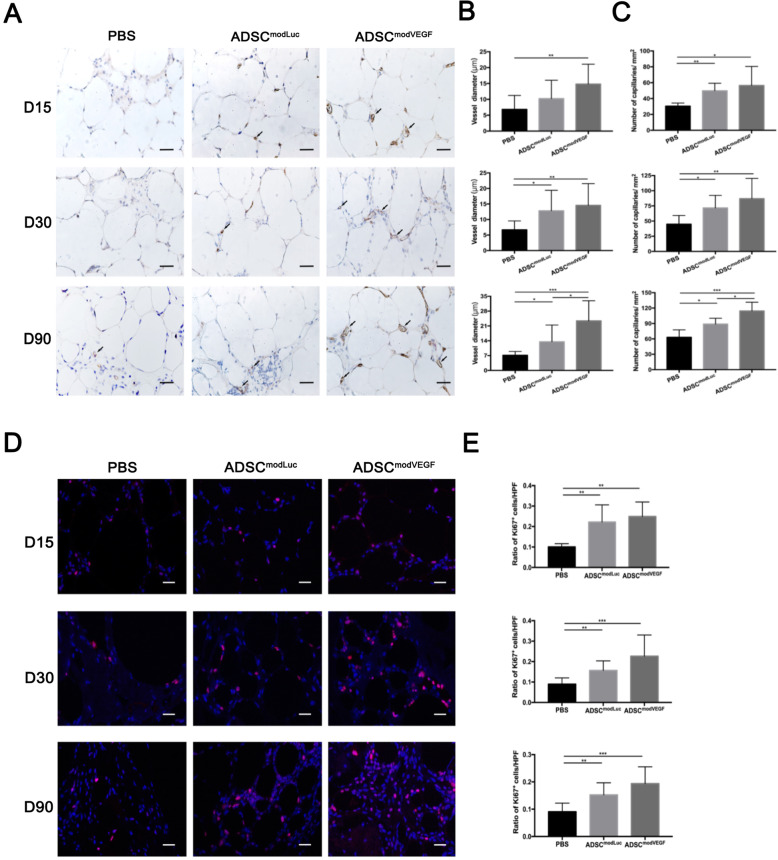
Histological evaluation of angiogenesis and cell proliferation in fat grafts. **a** CD31^+^ capillaries were stained and identified at 15, 30, and 90 days post-transplantation. Black arrowheads indicate blood vessels. **b**, **c** Quantitative analysis of **b** vessel diameters and **c** capillary densities among the different treatment groups. Note: Significantly larger vascular diameters and increased vessel densities were shown in the hADSC^modVEGF^ group. **d** Representative Ki67 stainings on paraffin sections of fat grafts at 15, 30, and 90 days post-transplantation. **e** Analysis of cell proliferation activity using Ki67^+^ stainings. Scale bars = 20 μm. Error bars showed means ± SD. (*p† < 0.05, **p† < 0.01, ***p† < 0.001)

To further evaluate the status of the grafted fat tissue, which had been treated with modVEGF-engineered hADSCs, we harvested and stained the grafts at 15, 30, and 90 days post-transplantation to assess neovessel formation and levels of cell proliferation. Using anti-CD31 antibodies and performing staining and analysis, we found more capillaries were formed in both the hADSC cell-assisted fat graft treatment groups, as visible already at day 15 post-transplantation (Fig. 6a). Note that the effects from the ADSC^modVEGF^ group gave rise to the most abundant capillary densities, which was evident in stained sections (Fig. 6a). In addition, when compared to the ADSC^modLuc^ group, the ADSC^modVEGF^ group presented with significantly larger vessel diameters and higher capillary densities, which together indicated better nutrient supply to adipocytes, at 90 days after grafting (*p* < 0.05) (Fig. 6b, c). Moreover, by analyzing percentages of proliferating cells in the grafts following transplantation, we found that both hADSCs and modVEGF-engineered hADSCs significantly promoted proliferation over the PBS control group. However, the ADSC^modVEGF^ group presented with the highest levels of cell proliferation at all time points studied (Fig. 6d, e). These findings suggest that transplantation of modRNA-engineered hADSCs induced neo-angiogenesis and increased cellular proliferation at levels significantly higher than those stemming from the non-modified hADSC-assisted fat transplant group. These findings suggest that the fusion of this molecular therapy together with cell-assisted fat grafts enhanced the composition of the grafted tissue in vivo.

### modVEGF-engineered hADSCs reduce the long-term absorption rate of grafted fat via alleviating fibrosis, apoptosis, and necrosis

**Fig. 7:**
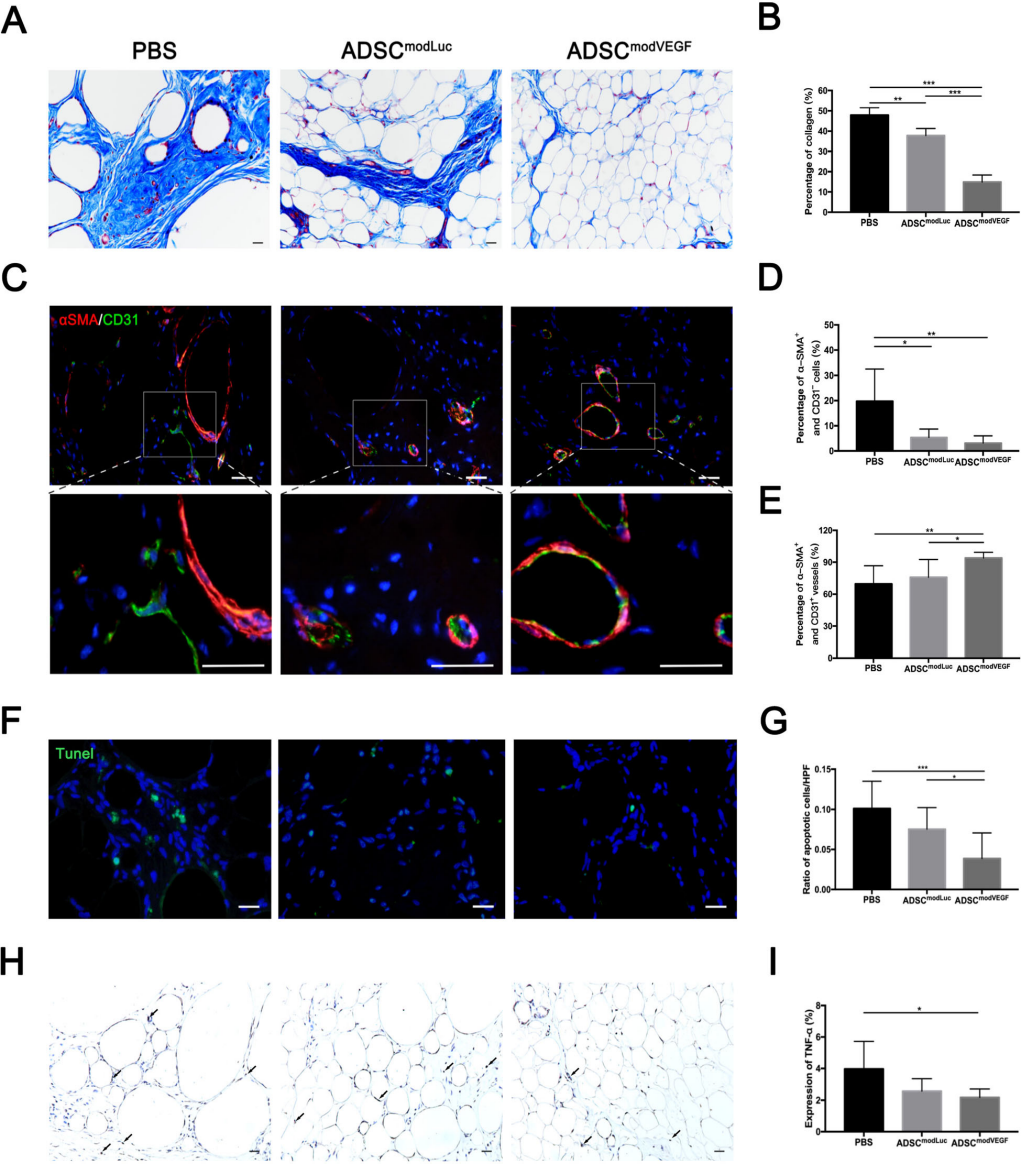
modVEGF-engineered hADSCs reduce levels of fibrosis, apoptosis, and necrosis in fat grafts. Fat grafts were harvested at 90 days after grafting for detailed molecular assessment. **a** Representative Masson’s trichrome staining of fat grafts. **b** Quantification of collagen (% area). Fibrotic areas are highlighted in blue with regions of extensive collagen deposition. **c** Representative immunostainings of α-SMA and CD31 shows mature blood vessels. **d** Analysis of α-SMA^+^ and CD31^−^ adipocytes (%). **e** Analysis of α-SMA^+^ and CD31^+^ vessels (%). Note: the hADSC^modVEGF^ group presented the least fibrotic tissue and gave rise to the most SMA^+^ capillaries, an indication of mature blood vessel formation. **f** Visualization of TUNEL^+^ cells indicates apoptotic activity. **g** Quantification of apoptotic cells. **h** Representative photomicrographs of TNF-α^+^ stainings. Black arrowheads practically indicate necrotic cells. **i** Analysis of TNF-α^+^ area (%). Scale bars = 20 μm. Error bars showed means ± SD. (*p† < 0.05, **p† < 0.01, ***p† < 0.001)

As the hADSC^modVEGF^ group presented with prominent beneficial effects, we next wanted to further investigate the long-term stability of these fat grafts at 90 days following transplantation. Morphological assessment of the grafts using Masson’s trichrome staining and analysis revealed that the ADSC^modVEGF^ group presented with significantly less fibrotic tissue among the three treatment groups, which is quantified in Fig. 7a, b (*p* < 0.001). The percentages of α-SMA^+^CD31^−^ cells represent the fibrotic adipocytes (Fig. 7c, d). The ADSC^modVEGF^ group maintained the healthiest looking adipose tissue, as blood vessels generated from this group were predominantly α-SMA^+^CD31^+^, demonstrating the ability for this treatment group to form more mature vessels and less fibrotic cells (Fig. 7c, e). TUNEL stainings revealed that there was a significant difference in levels of apoptosis between the ADSC^modLuc^ and the ADSC^modVEGF^ groups (*p* < 0.05) (Fig. 7f, g). Moreover, the ADSC^modVEGF^ group, but not the ADSC^modLuc^ group had significantly lower ischemia-induced necrosis when compared to the PBS group, as depicted by TNF-α levels (*p* < 0.05) (Fig. 7h, i). hADSCs engineered with modVEGF were capable of attenuating ischemia-induced fibrosis, apoptosis, and necrosis over a long-term follow-up and as such enhanced fat graft survival.

### modVEGF-engineered hADSCs enhance viability, retention, and in vivo differentiation to support a long-term survival of fat grafts

**Fig. 8:**
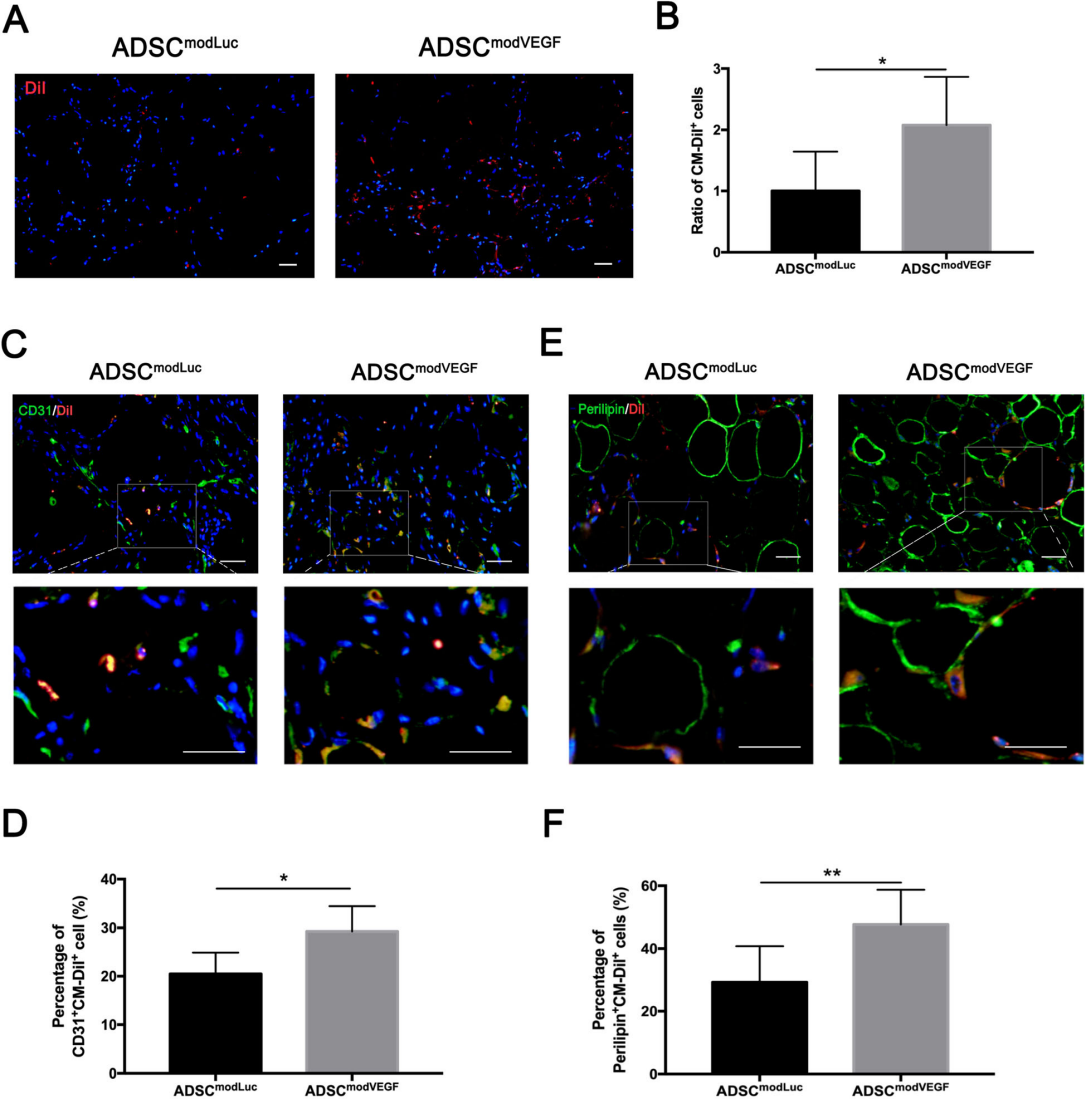
modVEGF-engineered hADSCs retain higher implantation viability and differentiation capacity to support a long-term survival of fat grafts. hADSCs engineered with modLuc or modVEGF were labeled with CM-DiI before mixing with fat and undergoing transplantation. Fat grafts were harvested at 90 days after grafting for histological evaluation. **a** Representative images of CM-DiI-labeled hADSCs in fat grafts. **b** Ratio of CM-DiI-labeled hADSCs. **c** Representative immunostainings of CD31- and CM-DiI-labeled cells identify vascular endothelial cells differentiated from hADSCs. **d** Percentage of CD31^+^ and CM-DiI^+^ cells in total CM-DiI^+^ cells (%). **e** Visualization of perilipin^+^ and CM-DiI^+^ cells indicates adipocytes differentiation activity from hADSCs. **f** Percentage of perilipin^+^ and CM-DiI+ cells in total CM-DiI^+^ cells (%). Note: the hADSC^modVEGF^ group presented with more viable hADSCs in fat grafts and gave rise to more capillaries and differentiated adipocytes. Scale bar = 20 μm. Error bars showed means ± SD. (*p† < 0.05, **p† < 0.01)

In order to trace and explore the fate of modRNA-engineered hADSCs, we employed CM-DiI labeling and histologically assessed fat grafts after a long-term transplantation in vivo. Simple histological analysis revealed more CM-DiI-labeled hADSCs had survived in the ADSC^modVEGF^ group at 90 days following grafting (Fig. 8a, b) (*p* < 0.05) when compared to the ADSC^modLuc^ control group. To determine the detailed fates of hADSCs, we stained sectioned grafts with a green fluorescein-labeled CD31 or perilipin to distinguish vascular endothelial cells and adipocytes derived from the exogenous CM-DiI-labeled hADSCs (Fig. 8c–f). Merging of the red fluorescence of CM-DiI with the green fluorescence of CD31 revealed a large portion of hADSCs treated with modVEGF took on an endothelial cell fate (Fig. 8c, d) (*p* < 0.05). Furthermore, an increased quantification of yellow adipocytes labeled with CM-DiI^+^ and green fluorescein-Perilipin^+^ was detected in the ADSC^modVEGF^ group, indicating better induction to mature adipocytes (Fig. 8e, f) (*p* < 0.01). Together, these results indicate hADSCs treated with modVEGF mRNA can better maturate into adipocytes and vascular endothelial cells, which ultimately enhance the long-term retention of transplants.

## Discussion

Herein, we report the first study emphasizing the therapeutic potential of combining hADSCs with modRNA technologies in order to reduce absorption rates in a humanized fat transplantation model.

At the early stage of fat grafting, there is no blood supply in grafts and the only source of nutrients is limited to the plasma surrounding the grafts [7]. As reported, timely and adequate revascularization is crucial for the survival of fat grafts, particularly in patients whom undergo large-volume fat transplants [37, 38]. Over the years, many investigators have reported the promise of hADSCs for cytotherapy via enhancing angiogenesis. hADSCs are a type of mesenchymal stromal/stem cell (MSC) arising from adipose tissue; meanwhile, other sources of MSCs include those isolated from the bone marrow (BMSCs), umbilical cord blood (UCB-MSCs), muscle (MDSCs), and dental pulp (DPSCs) [13]. Compared to the other populations of MSCs, several key advantages to working with hADSCs include the ease of isolation with minimal donor discomfort, as well as a higher quantity of collected cells (i.e., approx. 500-fold more MSCs are obtained in fat as opposed to BMSCs) [39, 40]. It has also been shown that hADSCs have more robust differentiation capabilities and as such may be better suited for treating a wider spectrum of ischemic injuries [33, 40–46]. Therefore, hADSCs have been exploited as a biological tool for angiogenic therapy, especially for tissue reconstruction, combating ischemia-related diseases, wound healing, and immune-regulation [26, 47–49].

Although ADSC-assisted fat transplants have been regarded as an optimal option to promote graft survival, previous studies observed hADSCs undergoing cell death prematurely and thus failed to induce timely and adequate angiogenesis post-transplantation [50–52]. VEGF, as a naturally secreted paracrine factor of hADSCs, directly promotes angiogenesis; however, one could speculate that the low levels of endogenously produced and secreted VEGF in these cells are insubstantial for a long-term vascularization [53, 54]. Fusing hADSC biologics with other cell-free-based technologies, such as recombinant human (rh) proteins or DNA vectors, are worthy alternatives to more effectively induce a long-term and stable angiogenesis. In our studies, we did not pursue such activities as VEGF rh proteins are used at supraphysiological doses to circumvent their short half-life, the results of which have led to the formation of leaky blood vessels and negative side-effects [20, 55]. On the other hand, DNA-based methods to engineer VEGF-secreting hADSCs would induce genomic integration and force long-term expression, a potential risk for systemic safety [16, 56]. Intriguingly, the significant advantages of a modRNA-based therapeutic modality are many, as highlighted in previous reports [17–20]. For example, the delivery and expression kinetics of modRNAs are met with high biocompatibility and low immunogenicity (Figs. 1and 2) [20, 23]. In addition, modRNA constructs are synthetically derived and can therefore be produced quickly and in large-scale format for applied and pre-clinical research in animal models of disease [57, 58]. Furthermore, the forced expression of VEGF protein from modRNA-engineered cells can exhibit burst-like therapeutic expression levels of VEGF protein in vivo before the loss of transplanted cells caused by initial ischemia, which has been reported (Fig. 1g and 4b) [23, 56].

Previous research reported that viable adipocytes of grafted fat were dramatically decreased as early as 24 h after transplantation, due to ischemic damage [7]. In our study, we found that modVEGF-engineered hADSCs were less prone to ischemic injury through mechanisms involving adequate vascularization and relieved hypoxic conditions (Fig. 1h and Fig. 4b, c). The transient yet abundant VEGF-A protein secreted from the modVEGF-engineered hADSCs was found to not only directly contribute to enhanced viability of hADSCs (Fig. 2a), but also increase the capacity for a long-term survival and better promote the in situ cell-fate potential (Fig. 3d, e and Fig. 8). We further revealed that the modVEGF-engineered hADSCs could stimulate neovascularization in a more favorable manner and induce the retention of more viable grafted cells enhancing the regenerate as made evident by the presence of expanded adipocytes (Fig. 5). Moreover, the ADSC^modVEGF^ group gave rise to significant vascularization and cell proliferation, seemingly persistent at 90 days post-transplantation (Fig. 6). In addition, modVEGF-engineered hADSCs could alleviate cell fibrosis, apoptosis, and necrosis over a long-term follow-up post-transplantation, which provides a strong case for the employment of hADSCs combined with modRNA technologies in the treatment of ischemic diseases (Fig. 7). Furthermore, previous studies have highlighted VEGF protein acting as a critical modulator of the tissue fibrosis and enhanced VEGF secretion potentially preventing/limiting apoptosis and necrosis under hypoxic conditions by activating the PI3K/Akt, TGF-β, or Notch pathways [59–61]. In this regard, it would be of interest to further evaluate mechanisms of modVEGF-engineered hADSCs on graft survival in our model. Exosomes isolated from hADSCs in hypoxic culture have also been reported to provoke a positive response regarding the survival of grafts and enhancing angiogenic ability [15]. Whether or not MSCs engineered with modRNA could enhance the beneficial effects seen by applications with exosomes is a worthy outlet worth exploring.

**Fig. 9:**
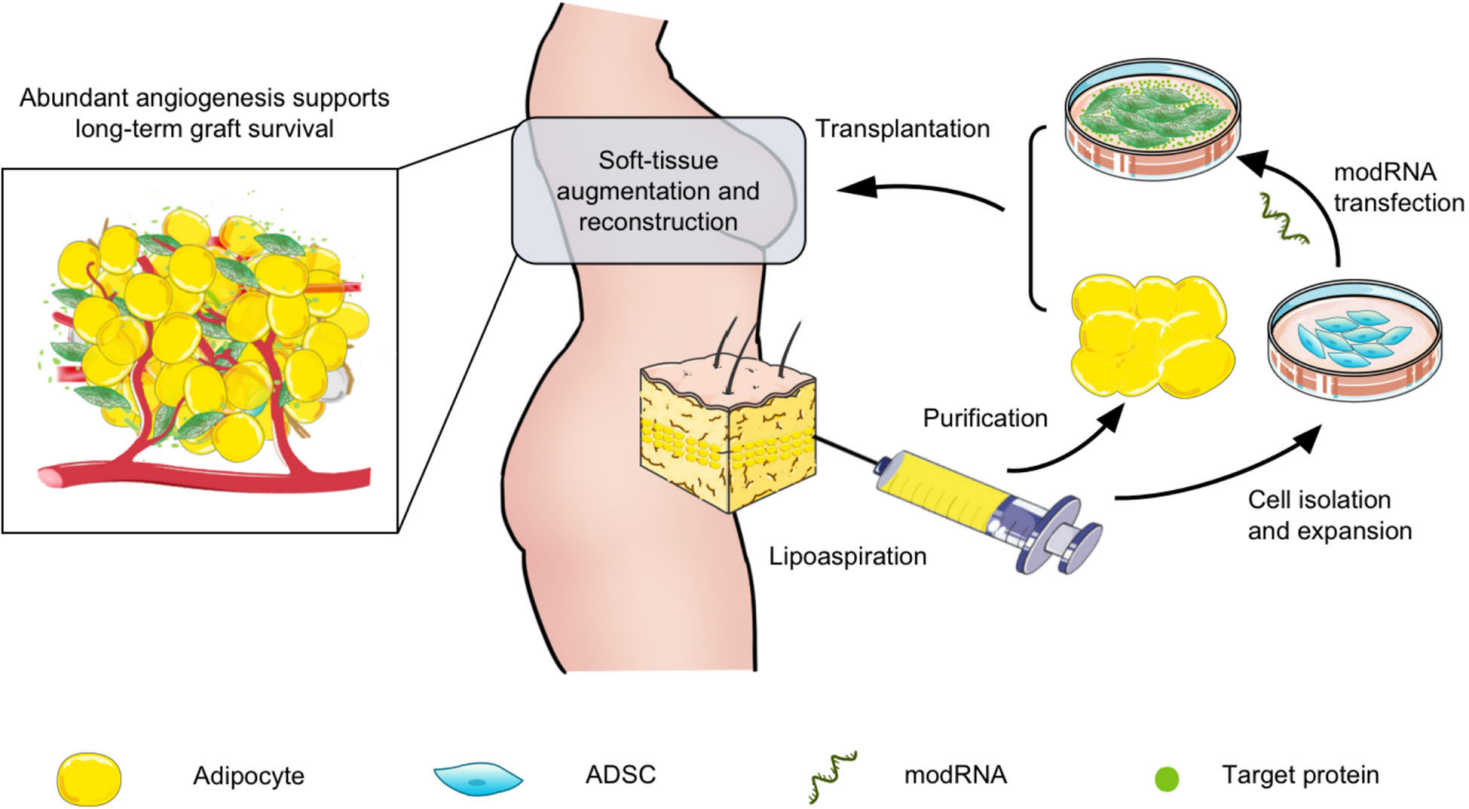
A novel combined model of hADSCs engineered with modRNAs could be considered as a potential treatment to promote angiogenesis and graft survival for clinical soft-tissue augmentation and reconstruction

Consistent with reported studies, we found no malignancy resulting from the hADSCs or modVEGF-engineered hADSCs applied in this study [10, 22]. Additionally, we provide novel support for effective therapies of modVEGF and broadened the application of modRNAs to tissue transplantation. As indicated in Fig. 9, we believe our therapeutic approach provides an optimal alternative to stem cell-assisted treatment in tissue transplantation and in regenerative medicine. In theory, stem cells could be easily harvested, carefully manipulated with modRNAs, and applied to the ischemic tissue to preserve cellular function and promote survival. Due to the safety and effectiveness of hADSCs and modRNAs, the novel combined model of hADSCs engineered with modRNAs could be considered as a potential treatment in the clinic for grafting and ischemic injuries.

## Conclusion

Our study highlights the optimal therapeutic potential of stem cells combined with modRNA technologies in tissue grafting. Importantly, results stemming from our study indicate that combining modRNA technologies with hADSCs dramatically enhances the therapeutic activity of the cells. Particularly, when applied to fat grafting, modVEGF-engineered hADSCs significantly improved the retention of fat grafts over naíve hADSCs through proangiogenic and pro-proliferative responses. Furthermore, this combinatorial treatment of hADSCs with modVEGF negatively regulated levels of fibrosis, apoptosis, and necrosis in fat grafts. This concept of a combinatorial treatment approach that merges cell-based and cell-free therapies has great clinical promise and could be a therapeutic strategy for treating wound defects, injuries stemming from vascular damage, or autoimmune disorders such as Graft-Versus-Host-Disease (GVHD).

## Supplementary Information

**Additional file 1.**

## Data Availability

The datasets used and/or analyzed during the current study are available from the corresponding author on a reasonable request.

## Abbreviations

hADSCs: Human adipose-derived stem cells
modRNA: Modified mRNA
VEGF-A: Vascular endothelial growth factor A
modVEGF: VEGF-modified mRNA
modLuc: Luciferase-modified mRNA
MSCs: Mesenchymal stromal/stem cells
BMSCs: Bone marrow-derived MSCs
T2DM: Type 2 diabetes mellitus
CLI: Critical limb ischemia
HUVECs: Human umbilical cord vein cells
ISCT: International Society for Cellular Therapy
H&E: Hematoxylin & eosin
TUNEL: Terminal deoxynucleotidyl transferase-mediated dUTP nick end-labeling
HIF-1α: Hypoxia-inducible factor 1-alpha
TNFα: Tumor necrosis factor alpha
αSMA: Alpha smooth muscle actin
