# Supplementary material for: Human adipose-derived stem cells enriched with VEGF-modified mRNA promote angiogenesis and long-term graft survival in a fat graft transplantation model
Source: Stem Cell Res Ther. 2020 Nov 19;11:490. doi: 10.1186/s13287-020-02008-8 (PMC7678328; doi:10.1186/s13287-020-02008-8)
Supplement: Supplementary file 1 — Additional file 1. [file 13287_2020_2008_MOESM1_ESM.docx]

**Supplemental Figures**


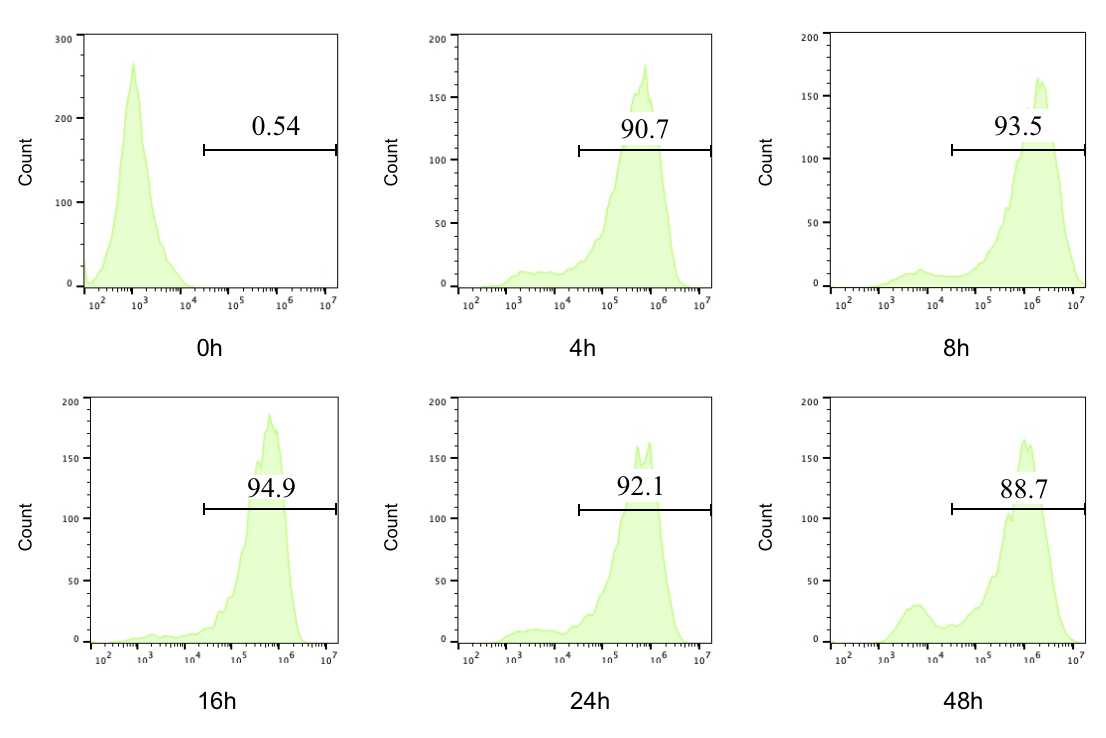


**Figure S1 Efficacy of modGFP transfection in hADSCs**

Transfection efficiency and kinetics of modGFP in hADSCs. Representative flow cytometry images of transfection efficiency pre-transfection (0h) and at 4, 8, 16, 24 and 48 h post-transfection.

**
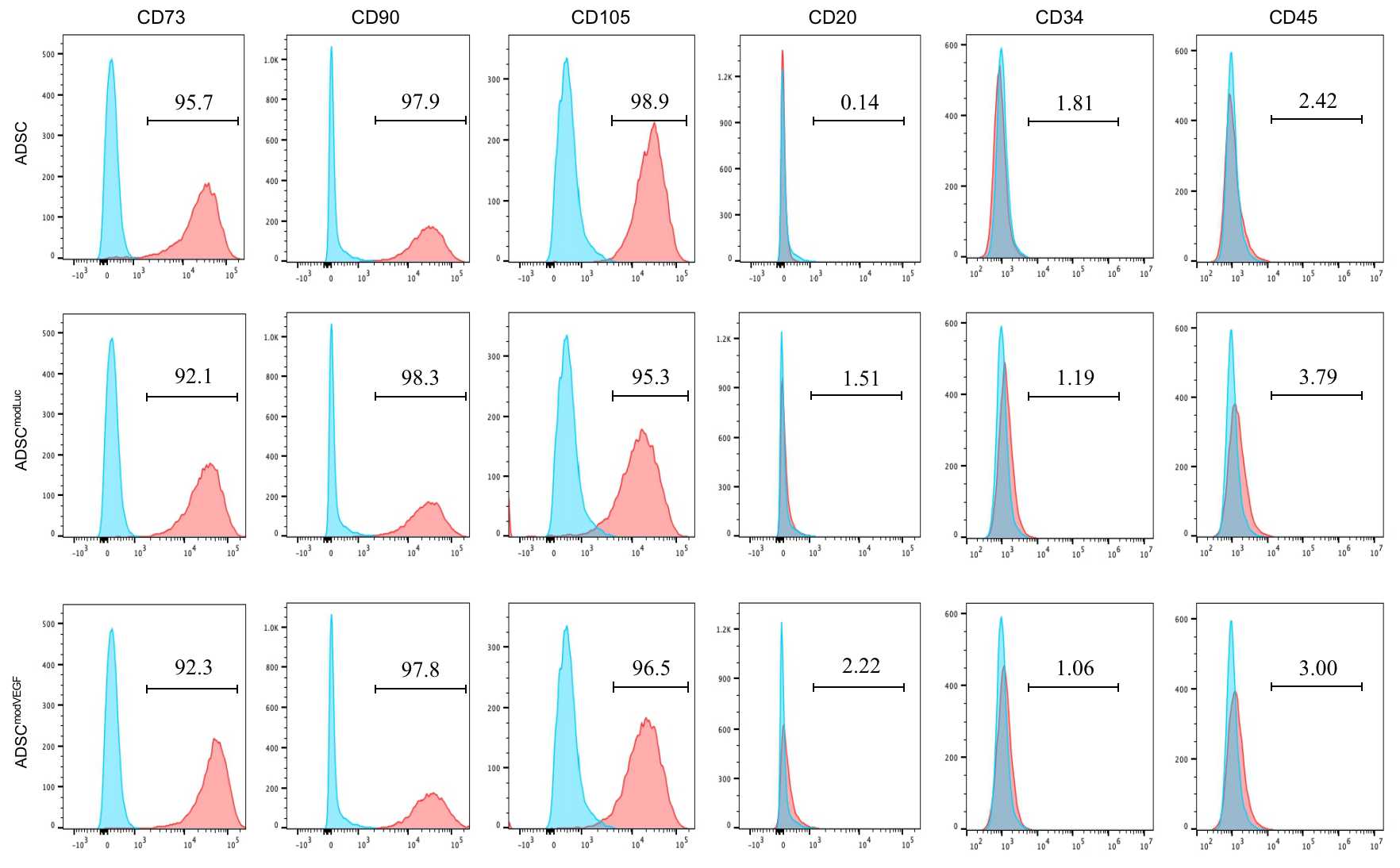
**

**Figure S2 Cell surface marker expression in modRNA-engineered hADSCs**

Representative flow cytometry images showed cell surface marker expression of hADSCs. hADSCs in all three groups revealed positive expression for CD73, CD90 and CD105; in contrast, negative expression (< 5%) of the hematopoietic lineage markers CD20, CD31 and CD45. Note: Surface marker expression was unaltered between naive hADSCs and modRNA-engineered hADSCs at 24 h after transfection.


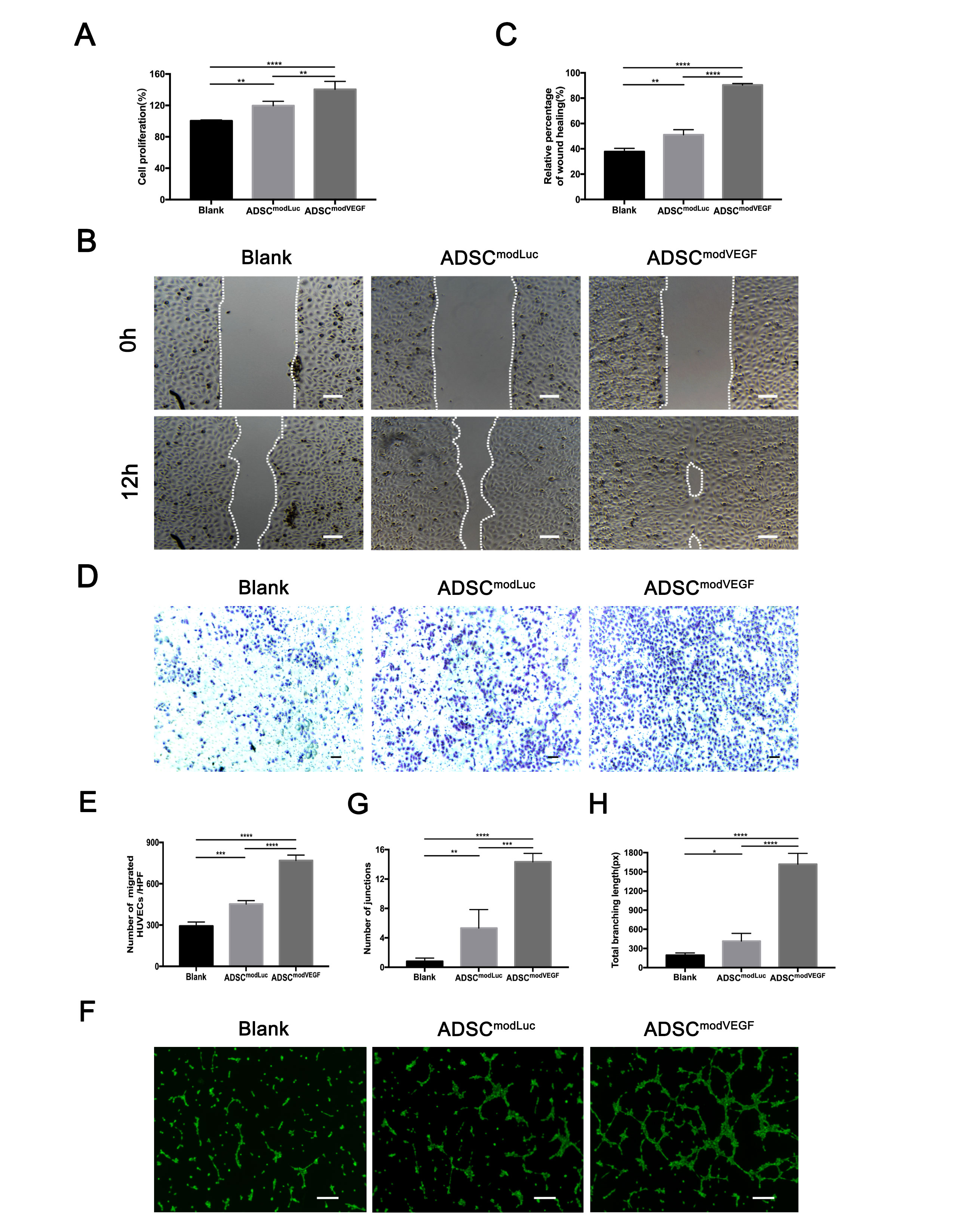


**Figure S3 Cultured medium from modVEGF-engineered hADSCs enhances HUVEC proliferation, migration and tube formation.**

HUVECs were treated with conditioned medium from modVEGF-engineered hADSCs or controls 24 h after transfection. (A) HUVECs proliferation was assessed using the CCK8 counting kit and the percentage of optical density values relative to control was calculated. (B-C) HUVECs migration was evaluated using a scratch test assay and the relative percentage of wound healing (at 12 h post injury) was quantified. (D-E) HUVECs invasion was evaluated using transwell assay and numbers of migrated cells identified by crystal violet (at 12 h) was measured. (F) After 6 h incubation, HUVECs were stained with Calcein-AM to assess levels of tube formation. (G-H) Total branching length and the number of junctions were counted. Scale bar = 100 μm. Error bars showed means ± SD.(n=3; *p†<0.05, **p†<0.01, ***p†<0.001, ****p†< 0.0001).

**Supplemental Materials and Methods**

**Flow cytometry**

The transfection efficiency of modGFP in hADSCs and the phenotypic profile of hADSCs were examined by flow cytometry as the main text described. The results and the representative pictures were analyzed by FlowJo software (Ashland, OR).

**Effects of modVEGF-engineered hADSC conditioned medium on human vascular endothelial cells (HUVECs)**

The characteristics of HUVECs after modVEGF transfections were examined as previously described [23]. HUVECs were treated with 50% (v/v) conditioned medium from either an acellular group (Blank), modLuc-engineered hADSCs (ADSC^modLuc^) or modVEGF-engineered hADSCs (ADSC^modVEGF^) to evaluate the effects on proliferation, migration and tube formation. Briefly, the proliferation of HUVECs were determined by the CCK8 (Dojindo, Kumamoto, Japan) following the recommended protocol and data are presented as the ratio of the O.D. value relative to the control group. To determine the effects of the conditioned media on cell migration, HUVECs at 90% confluency were scratched with a sterile yellow pipette tip and left to incubate in the conditioned medium mentioned above. Images at 0 and 12 h were compared using ImageJ software (NIH, Bethesda, MD, USA). The tube formation assay was conducted by first culturing the HUVECs on growth factor-reduced Matrigel (BD Biosciences) pre-coated plates. Next the HUVECs were cultured in the aforementioned conditioned medium above for 6 h, after which the cells were stained with Calcein-AM solution (Yeason, Shanghai, China) and imaged under fluorescence microscopy (Olympus BX51, Japan). The number of junctions and total branching length were calculated using ImageJ software (NIH). All experiments were performed in triplicates.
